# Association of Magnesium Deficiency and Reduction in Blood Pressure After Chemotherapy in Previously Hypertensive Cancer Patients: The Role of Chemotherapy and Magnesium Levels

**DOI:** 10.3390/medicina61081357

**Published:** 2025-07-26

**Authors:** Aurora Soldado, Kevin Doello, Jose Prados, Cristina Mesas, Consolacion Melguizo

**Affiliations:** 1 Medical Oncology Service, Virgen de las Nieves Hospital, 18016 Granada, Spain; aurora.soldado.sspa@juntadeandalucia.es (A.S.); kdoello@ugr.es (K.D.); 2 Instituto de Investigación Biosanitaria de Granada (ibs.GRANADA), 18012 Granada, Spain; melguizo@ugr.es; 3 Institute of Biopathology and Regenerative Medicine (IBIMER), Center of Biomedical Research (CIBM), University of Granada, 18100 Granada, Spain; 4 Department of Anatomy and Embryology, Faculty of Medicine, University of Granada, 18071 Granada, Spain

**Keywords:** hypertension, normotension, cancer, cisplatin, magnesium

## Abstract

*Background and Objectives*: A commonly observed phenomenon in outpatient oncological patients is the appearance of hypotension not attributable to other causes in hypertensive patients undergoing oncological treatment. Once antihypertensive treatment is discontinued, patients remain normotensive after the oncological treatment ends. The objective of this research is to analyze our experience with this phenomenon and try to provide an explanation. *Materials and Methods*: A retrospective case-control study was conducted with a total sample of 302 hypertensive oncological patients, with cases presenting symptomatic hypotension and controls not. Descriptive and inferential statistics were performed, with the latter focusing on studies by Odds Ratio, Chi-square, Z test for comparison of two proportions, and multivariate regression. *Results*: Regarding the results obtained, it is noteworthy that in both the univariate and multivariate models, treatment with cisplatin showed statistical significance (Univariate, OR 3.06 (CI 1.82–5.11). Z 4.45, *p* < 0.0001; multivariate, *p* < 0.001, Nagelkerke R2 74.8%). Cisplatin treatment and the study phenomenon were correlated with magnesium levels (Chi-square 8.2, *p* = 0.017), relating hypotension to hypertensive patients with low magnesium levels. *Conclusions*: CDDP treatment is associated with hypotension or normotension in previously hypertensive cancer patients. This may be related to peripheral vascular fragility induced by oncological drugs, leading to reduced vascular resistance. Although magnesium deficiency is generally linked to hypertension, chemotherapy-related shifts in magnesium levels due to impaired renal handling may play a role. These findings may help improve the understanding of blood pressure regulation in oncology patients.

## 1. Introduction

Among cancer patients, hypertension prevalence reaches 37%, making it not only a common comorbidity but also the most frequent adverse event. Since 1975, studies have examined the association between hypertension and cancer. While data remain inconsistent, it appears that the relationship between metabolic syndrome and cancer primarily hinges on hypertension and abdominal circumference. Nevertheless, confounding factors such as surveillance bias, aging, shared risk factors (inactivity, smoking, diabetes), and chronic inflammation (e.g., from atherosclerosis) may underlie both conditions. Notably, overactivation of Wnt and AMPK pathways, central to inflammation, has been observed in both hypertensive and cancer patients [1].

Chemotherapy-induced hypertension can arise from both direct and indirect effects, such as kidney damage. VEGF inhibitors with tyrosine kinase inhibitor (TKI) activity are closely associated with hypertension. Other agents like cisplatin, cyclophosphamide, bendamustine, and busulfan cause vascular and renal toxicity. Gemcitabine and proteasome inhibitors (e.g., bortezomib, carfilzomib) can induce hypertension via thrombotic microangiopathy. Adjuvants like NSAIDs, corticosteroids, erythropoiesis-stimulating agents, and calcineurin inhibitors also contribute. Additionally, cranio-cervical radiotherapy can impair baroreceptor function, leading to either hypo- or hypertension [2]. Hypertension in oncology patients may increase the risk of cardiotoxicity and could lead to treatment interruption. Anticancer chemotherapy can induce myocardial damage, leading to hypotension and potential magnesium deficiency. This damage arises from various mechanisms, including direct toxicity to heart muscle cells, disruption of cellular energy production (metabolism), and oxidative stress. These effects can manifest as heart failure, arrhythmias, and other cardiovascular complications [3].

While most studies focus on the high incidence of hypertension, arterial hypotension (<90/60 mmHg) is also frequently encountered during the clinical course of cancer. Symptoms such as dizziness, fainting, weakness, fatigue, blurred vision, confusion, and cold sweats result from decreased cerebral perfusion. Some individuals may remain asymptomatic despite low blood pressure. Severe hypotension or shock can compromise vital organs and represents a potentially life-threatening condition, especially in oncology patients [4].

The exact incidence and prevalence of hypotension in cancer patients remain undetermined due to limited studies. Moreover, symptoms are often misattributed to cancer or its treatment, complicating accurate identification.

Several factors—including the disease itself, secondary complications like infections, bleeding, thrombosis, and many antineoplastic drugs—alter hemodynamics, sympathetic tone, renal function, and plasma electrolyte levels. The common pathophysiological mechanism in these processes is often hypovolemia. Nonspecific symptoms make diagnosis challenging in both outpatient and inpatient settings. Reduced tissue perfusion manifests as fatigue, thirst, cramps, and dizziness. In severe cases, abdominal or chest pain and confusion may occur [5].

Additionally, certain tumors—such as carcinoids and pheochromocytomas—directly affect the endocrine system, altering hormone levels that regulate blood pressure (e.g., serotonin, catecholamines). Tumor growth may compress vascular structures, impacting blood pressure. Malnutrition and cachexia, common in advanced-stage cancer patients, also contribute to hypotension [6].

Systemic antitumor treatment may significantly affect blood pressure regulation. Some chemotherapeutic agents directly impair the cardiovascular system. For instance, anthracyclines and doxorubicin are well-documented as inducing cardiotoxicity and weakening myocardial function. Myelotoxicity may reduce red blood cell production, causing hypoxia and lowered blood pressure. Systemic inflammatory responses can also lead to vasodilation and reduced vascular resistance. Cases of non-allergic hypotension have been reported with irinotecan [7].

Susceptibility to chemotherapy-induced hypotension varies by drug type, dosage, duration, and patient-specific factors. Thus, monitoring and managing side effects is crucial during treatment. Vasovagal syncope, an exaggerated autonomic response, results in transient cerebral hypoperfusion and loss of consciousness, triggered by pain, fatigue, heat, or emotional stress. Cardiovascular impact from chemotherapy, radiotherapy, or immune checkpoint inhibitors can increase the risk of hypotension and syncope. Central nervous system tumors or mediastinal masses may also impair autonomic function. Moreover, antiemetics and opioids can affect autonomic regulation [7].

Interestingly, certain antihypertensive drugs such as angiotensin II receptor blockers (ARBs) and angiotensin-converting enzyme inhibitors (ACEIs) may have antitumor effects. Laboratory and animal studies suggest they may inhibit cancer cell proliferation, angiogenesis, and metastasis. However, further research is needed to determine their clinical relevance. Their role in modulating inflammation and angiogenesis links them to cancer development and progression. ARBs can reduce growth factor and proinflammatory cytokine production, potentially altering the tumor microenvironment and immune response. Calcium channel blockers may influence tumor perfusion and treatment response [8].

Magnesium deficiency (hypomagnesemia) is a common finding in cancer patients undergoing chemotherapy, largely due to the effects of both the malignancy and the chemotherapeutic agents. These treatments can impair gastrointestinal absorption, increase renal magnesium losses, or alter intracellular distribution [9,10,11]. Although magnesium deficiency is typically associated with hypertension [12,13,14], its broader cardiovascular effects in the context of chemotherapy—including potential influences on vascular tone and reactivity—are not fully understood and warrant further investigation [10,15].

A phenomenon that is seen with some frequency in outpatient cancer patients is the fact that hypertensive patients suffer symptomatic hypotension, being forced to stop antihypertensive treatment during cancer treatment. When they finish it, they remain normotensive without the need for antihypertensive medication.

Therefore, this study aims to investigate the causes of hypotension and subsequent sustained normotension in previously hypertensive oncology patients.

## 2. Materials and Methods

The study included all hypertensive oncology patients treated at the Medical Oncology service of Virgen de las Nieves University Hospital who had experienced episodes of symptomatic hypotension and required a reduction or discontinuation of antihypertensive treatment during the period from 2017 to 2023. The controls were a proportionally randomized sample of hypertensive patients treated in the same service during the same period who remained hypertensive, even if they had experienced isolated symptomatic hypotension justified by another acute cause (hospitalization, infection, dehydration, etc.), and who continued to require antihypertensive treatment after returning to their baseline condition.

A retrospective cases and controls study was developed. A total of 302 patients participated in the study, comprising 156 cases that were paired with controls with similar characteristics in a 1:1 ratio. However, 10 control patients withdrew informed consent, so that controls were 146. Data were collected retrospectively from the secondary source “Diraya” the Unique Health History. The data were subsequently anonymized. The search criterion for the primary variable studied (symptomatic hypotension) was the word “dizziness.” Then, through a thorough review of each patient’s history, focusing particularly on whether episodes of hypo/normotension led to a reduction or discontinuation of antihypertensive treatment, patients were included and classified into cases or controls group.

More exactly, the patients were randomly obtained by aleatory numbers table and the inclusion criteria were all patients with active cancer receiving chemotherapy who had experienced episodes of symptomatic hypotension. The exclusion criterion was to exclude cancer patients who had not received systemic treatment. Once included, the patients were stratified into cases and controls based on the aforementioned criteria. There were age and sex matching between cases and controls where possible. All study variables, such as sex, age, chemotherapy, sustained hypotension, time to hypotension, type of antihypertensives, number of antihypertensives, treatment line, tumor stage, type of tumor, morphine use, magnesium serum levels, were obtained from health history.

Afterwards, a descriptive analysis was performed to evaluate the prevalence, frequency, and distribution of the study population. Following this, an inferential analysis was conducted to compare relevant variables between the two groups. The odds ratio, the Z test and Chi-square test were applied, considering a *p*-value < 0.05 as statistically significant. Using the Statistical Package for Social Sciences (SPSS, v28.0.1 software), a Multiple Logistic Regression Model was developed to predict the probability of having hypotension and/or normotension based on potentially explanatory variables. This latter statistical methodology was used to correlate the presence of hypotension/normotension with possible associated factors, helping to eliminate confounding factors such as comorbidities, concomitant medication, type of chemotherapy, etc.

## 3. Results

**Figure 1:**
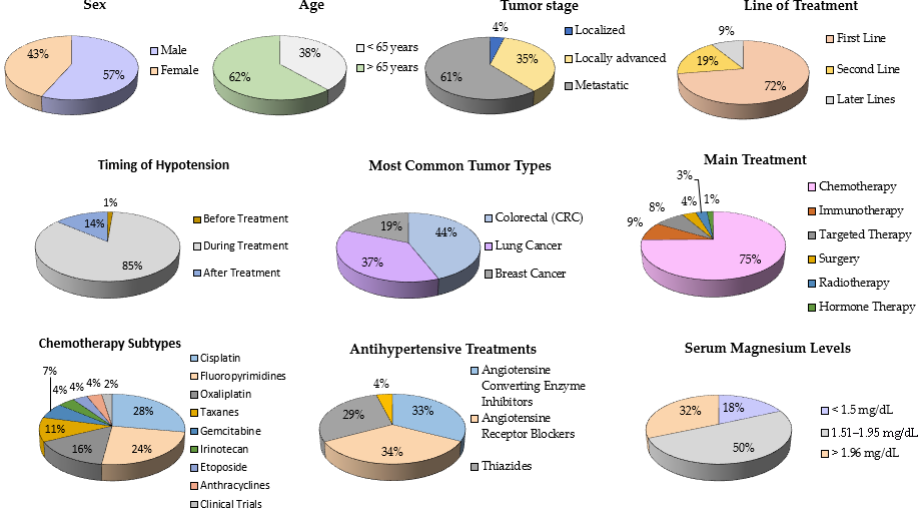
Representation of descriptive statistics on the oncological characteristics of the patients studied.

The study included 156 cases and 146 controls. In terms of descriptive statistics, the main characteristics of the population, such as sex and age, are distributed homogeneously; 43.4% women vs. 56.6% men and 38.3% young people (under 65 years) vs. 61.7% over 65 years. A total of 42.7% of the patients were undergoing opioid treatment. The majority of patients (60.6%) were in a metastatic stage, followed by 35.4% in a locally advanced stage, and only 4% in a localized stage. Overall, 72.5% of the patients received first-line treatments, 18.5% second-line treatments, and 8.8% received later treatments. The development of hypotension occurred mainly during treatment (84.7%), followed by 14% who developed it after treatment, and 1.3% before treatment. The most frequent tumors are colorectal cancer (26.5%), lung cancer (22.5%), and breast cancer (11.3%). Chemotherapy is the predominant treatment, used in 74% of the patients. Other treatments include immunotherapy (8.6%), targeted therapies (7.9%), surgery (3.6%), radiotherapy (2.9%), and hormone therapy (1.65%). Among the main chemotherapies, cisplatin (CDDP) is the most commonly used, accounting for 32.8% of patients. It is followed by fluoropyrimidines (28.8%), oxaliplatin (18.5%), taxanes (13.2%), gemcitabine (8.3%), CPT11 (5%), etoposide (4.3%), anthracyclines (4.3%), and clinical trials (3%). A total of 51% of patients were receiving treatment with angiotensin converting enzyme (ACE) inhibitors, 54% with angiotensin receptor blockers (ARBs), 45% with thiazides, and 6.3% with calcium antagonists, in various combinations among them. Overall, 50.4% of patients had mild/moderate hypomagnesemia, 31.3% had normomagnesemia, and 18.3% had very low magnesium levels, below 1.5 mg/dL (Figure 1).

The distribution of patients taking morphine is similar in patients with hypotension (cases: 54.7%) and those without it (controls: 47.7%). Hypotension is more common in patients receiving CDDP (69.7%) compared to those who are not (42.9%) (Table 1).

In terms of inferential statistics, univariant contingency tables were employed to apply the Z statistic and calculate odds ratio (OR), indicating the risk of hypertensive cancer patients no longer being hypertensive based on various exposures. In this case, a univariate association was obtained between symptomatic hypotension and age (OR 1.43 (CI 0.908–2.265). Z 2.1, *p* = 0.035) and between symptomatic hypotension (cases) and treatment with CDDP (OR 3.06 (CI 1.82–5.11). Z 4.45, *p* < 0.0001), the association being especially significant and intense in the latter case (Table 2).

Moreover, a multiple logistic regression model was then developed to examine the relationship between symptomatic hypotension and maintained normotension (cases) and several predictors. The final model showed strong statistical significance (*p* = 0.000) with a Nagelkerke R2 value of 74.8%, indicating a good fit for predicting symptomatic hypotension in cancer patients. Factors such as stage and certain treatments (CDDP, Carboplatin, Pemetrexed, Immunotherapy, Gemcitabine and Anthracyclines) were significant (*p* < 0.05), while the use of CDDP showed the strongest signification (*p* = 0.000). The number of antihypertensives was also relevant, though with a borderline *p*-value (*p* = 0.065), as well as the ingestion of morphs (*p* = 0.025) (Table 2).

Additionally, a significant association between CDDP treatment and lower serum magnesium levels was found. Patients treated with CDDP had a higher incidence of hypomagnesemia, while those not treated with it showed higher serum magnesium levels. Chi-square tests confirmed this association (Chi-square value: 8.2, *p* = 0.017). Among normotension/hypotension patients, both for patients who did not receive treatment with CDDP and those who did, mild or moderate hypomagnesemia predominates (42% in the first group and 65.2% in the second). A total of 35.8% of patients not treated with CDDP had normal values, while 24.2% of patients treated with the drug had normal levels. Severe low magnesium levels in the CDDP group corresponded to 10.6%, and in the not treated group, to 22.2% (Table 3).

## 4. Discussion

This study demonstrates that treatment with cisplatin is associated with a significant decline in blood pressure among patients with pre-existing hypertension. While both hypotension and magnesium deficiency have been observed in patients receiving anticancer chemotherapy, the exact relationship between these phenomena remains unclear and has not been extensively explored in the previous literature. Hypotension in this context is likely multifactorial, potentially involving chemotherapy-induced vascular or myocardial damage. Although magnesium deficiency is a common side effect of certain chemotherapies, its direct role in causing hypotension is not well established and warrants further investigation [16,17].

**Figure 2:**
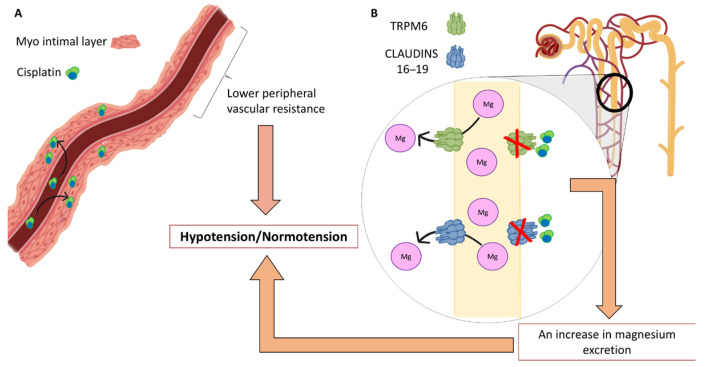
Summary of the main hypothesized mechanisms that explain normotension after chemotherapy treatment in hypertensive patients. (**A**) The possible effect of CDDP against myo-intimal vascular layer and vascular peripheral resistances. (**B**) Represents the possible effect of cisplatin in the decrease of magnesium renal reabsorption and in the increase of its urinary excretion.

The fact that CDDP was statistically significant, both in univariant and in the multivariant regression model, leads us to wonder to what degree and at what level it influences blood pressure. This agent has a direct impact on the function of blood vessels, potentially inducing vascular damage and endothelial dysfunction, leading to decreased peripheral vascular resistance and blood pressure. Recent studies in murine species have described, for example, its association with erectile dysfunction by affecting the smooth muscle content and endothelial function in the corpus cavernosum. Through staining, morphological changes and alterations in the proportion of smooth muscle to collagen were demonstrated, with a significant reduction in collagen content [18,19,20] (Figure 2). The study published by Stelwagen et al. demonstrates with a sample of 127 patients that CDDP induces chronic vascular damage after, and an accelerated vascular aging [21].

Cisplatin (CDDP) has been shown to downregulate the expression of TRPM6, a transporter responsible for renal magnesium reabsorption, resulting in increased magnesium excretion and subsequent urinary loss. This increased magnesium excretion is often accompanied by sodium and water loss, which may contribute to hypotension [22,23,24] (Figure 2). This mechanism could partially explain the association observed between cisplatin treatment and hypotension or normotension in patients with low serum magnesium levels. Furthermore, Lajer et al. reported that approximately 90% of patients receiving CDDP develop hypomagnesemia [10]. Suppadungsuk et al. demonstrated that intravenous magnesium supplementation can help prevent CDDP-induced nephrotoxicity [25].

Age was significant in univariate analysis but not in multivariate analysis, possibly because it acts as a confounding factor related to CDDP use, given that older patients (over 75 years) are less frequently prescribed cisplatin.

The main limitation of this study is its case-control design, which does not allow determination of the prevalence of sustained hypotension after chemotherapy in cancer patients. Therefore, prospective cohort studies are necessary to investigate this phenomenon more thoroughly.

## 5. Conclusions

This study found an association between hypotension and chemotherapy in previously hypertensive patients, potentially involving changes in magnesium homeostasis. While magnesium deficiency is more commonly associated with hypertension, chemotherapy-induced alterations in renal magnesium handling may contribute to complex hemodynamic effects, including hypotension, in certain clinical settings. Further investigation through randomized controlled trials and well-designed cohort studies is necessary to clarify the role of magnesium and other contributing factors in blood pressure changes observed during cancer treatment.

## Figures and Tables

**Table 1:** Inferential univariant studies developed in studied patients.

Variable	Cases (Yes)	Cases (No)	Controls (Yes)	Controls (No)	OR	IC	Z	*p*
Sex (male)	95	61	76	70	1.422	(0.890–2.27)	1.61	0.11
Age (up to 65 years)	66	90	49	95	1.43	(0.908–2.265)	2.1	0.035
Fluoropirimidines	50	106	37	109	1.39	(0.841–2.296)	1.34	0.177
Taxanes	20	136	20	126	0.926	(0.476–1.802)	0.31	0.75
Etoposide	10	146	3	143	3.265	(0.880–12.108)	1.75	0.072
Irinotecan	10	146	5	141	1.93	(0.644–5.792)	1.062	0.28
Pemetrexed	12	144	6	140	1.94	(0.710–5.324)	1.28	0.29
Gemcitabine	16	140	9	137	1.74	(0.744–4.070)	1.2	0.22
Carboplatin	18	138	20	126	0.822	(0.416–1.624)	0.25	0.82
Oxaliplatin	33	123	23	123	1.44	(0.78–2.58)	1.12	0.27
Cisplatin	69	87	30	116	3.06	(1.82–5.11)	4.45	0.0001
Anthracyclines	7	149	6	140	1.09	(0.36–3.34)	0.17	0.86
Immunotherapy	9	147	9	137	0.932	(0.36–2.41)	0.11	0.91
Biologicals	19	137	17	129	1.052	(0.524–2.11)	0.13	0.89
Clinical trials	3	153	6	140	0.46	(0.112–1.86)	0.64	0.552
Surgery	4	152	8	138	0.45	(0.13–1.54)	1.08	0.28
Angiotensine Converting Enzyme Inhibitors	84	72	70	76	1.26	(0.806–1.991)	1.04	0.89
Angiotensine Receptor Blockers	79	77	86	60	0.716	(0.454–1.128)	1.56	0.11
Thiazides	69	87	67	79	0.935	(0.594–1.472)	0.34	0.72
Calcium antagonists	10	146	9	137	1.043	(0.411–2.643)	0.147	0.88

**Table 2:** Multivariate regression study on the oncological studied patients.

Effect	Log-Likelihood	Chi-Square	*p*
Age	163.493	0.354	0.552
Sex	163.158	0.018	0.893
Type Of Tumor	170.174	7.034	0.071
Stage	170.815	7.676	0.053
Carboplatin	167.255	4.115	0.043
Fluoropyrimidines	166.669	3.529	0.060
Taxanes	166.358	3.218	0.073
Immunotherapy	167.327	4.187	0.041
Etoposide	164.207	1.067	0.302
Biologicals	165.636	2.496	0.114
Irinotecan	163.513	0.373	0.541
Pemetrexed	167.403	4.264	0.039
Gemcitabine	171.445	8.305	0.004
Clinical Trial	166.839	3.700	0.054
Radiotherapy	163.500	0.361	0.548
Surgery	167.575	4.435	0.035
Oxaliplatin	166.436	3.296	0.069
Anthracyclines	168.191	5.051	0.025
Cisplatin	179.145	16.006	0.0001
Treatment Line	168.695	5.555	0.235
Number of Antihypertensives	168.596	5.456	0.065
Angiotensine Receptor Blockers	164.856	1.717	0.190
Thiazides	163.140	0.000	0.994
Calcium Antagonists	166.019	2.879	0.090
Angiotensine Converting Enzyme Inhibitors	163.433	0.293	0.588
Morphine Use	168.131	4.991	0.025
Time Of Hypotension	200.444	37.304	0.000

**Table 3:** Chi-square table showing the relationship between CDDP treatment and magnesium blood levels.

Variable	Patients Without Cisplatin (%)	Patients with Cisplatin (%)	Chi-Square (χ^2^)	*p*-Value
Mild/Moderate Hypomagnesemia	34 (42%)	43 (65.2%)	8.2	0.017
Normal Magnesium Levels	29 (35.8%)	16 (24.2%)	-	-
Severe Hypomagnesemia (<1.5 mg/dL)	18 (22.2%)	7 (10.6%)	-	-

## Data Availability

Data available on request due to restrictions.
